# Superselective Transarterial Chemoembolization for Unresectable or “Ablation Unsuitable” Hepatocellular Carcinoma in the Caudate Lobe: A Real World, Single-Center Retrospective Study

**DOI:** 10.3389/fonc.2021.678847

**Published:** 2021-10-28

**Authors:** Liangliang Yan, Yanqiao Ren, Kun Qian, Xuefeng Kan, Hongsen Zhang, Lei Chen, Bin Liang, Chuansheng Zheng

**Affiliations:** ^1^ Department of Radiology, Union Hospital, Tongji Medical College, Huazhong University of Science and Technology, Wuhan, China; ^2^ Hubei Key Laboratory of Molecular Imaging, Wuhan, China

**Keywords:** transarterial chemoembolization, hepatocellular carcinoma, caudate lobe, overall survival, progression-free survival

## Abstract

**Objectives:**

To analyze the clinical outcomes of Transarterial chemoembolization (TACE) for unresectable or “ablation unsuitable” hepatocellular carcinoma (HCC) in the caudate lobe (CL) found at initial presentation in clinical practice.

**Methods:**

Fifty-eight patients with HCC-CL undergoing conventional TACE from January 2015 to January 2020 were enrolled in our medical center. Overall survival (OS), progression-free survival (PFS), tumor response rate and major complication rates were analyzed. Multivariate analyses for potential clinical and radiologic factors were performed by using the Cox proportional hazard model.

**Results:**

The median OS was 23 months (95%CI: 18.1-27.9), and the median PFS was 11 months (95%CI: 7.4-14.6). The 1-, 3-, and 5-years OS rates were 66.5%, 31.9% and 15.7%, respectively. The 0.5, 1-, and 3-years PFS rates were 60.3%, 44.5% and 6.3%, respectively. Objective response rate was 53.4% and disease control rate was 79.3%. The most serious complication was bile duct injury, with an incidence of 3.4%. Multivariable analysis revealed that total bilirubin, Barcelona Clinic Liver Cancer stage, nonselective chemoembolization and TACE session were four significant factors associated with OS.

**Conclusions:**

Superselective TACE treatment might be associated with better survival benefits in unresectable or “ablation unsuitable” HCC in the CL without macroscopic vascular invasion (MVI) and adequate liver function, compared with the non-selective TACE group, and should be considered as an important reliable therapy for surgeons and interventional radiologists.

## Introduction

Hepatocellular carcinoma (HCC) is a common malignancy, with incidence and mortality rates ranking sixth and third in the world, respectively (1). Cases of HCC arising or involving the caudate lobe (HCC-CL) are relatively rare, and its incidence is reported to be between 1.9% and 12.4% (2–7). Although the implementation of surveillance programs for high-risk populations and advances in imaging diagnostic technology have increased the diagnosis rate of HCC-CL (8, 9), about 80% of patients have tumors that are unresectable owing to either severe hepatitis-related cirrhosis or tumor invasion of the intrahepatic vessels (10). Surgical resection and percutaneous radiofrequency ablation (RFA) can be used as a curative treatment for HCC-CL (11, 12). However, resection of the HCC-CL is a challenging task for accomplished surgeons owing to the tumor’s deep location that is adjacent to the inferior vena cava and hepatic vein and narrow surgical margin (2, 3, 6, 13, 14). Radiofrequency therapy cannot be performed safely under certain circumstances, such as thermal injury of adjacent structure, heat sink effect (near major vessels), and limited tumor necrosis range (15). Although some radical treatments or combination treatments have favorable clinical benefits in several studies (16–18), the five-year recurrence rate of HCC is still high.

Therefore, intra-arterial therapy which causes tumor necrosis through the occlusion of blood flow and the slow release of chemotherapeutic drugs into tumors is the mainstay palliative treatment options recommended by the Barcelona Clinic Liver Cancer (BCLC) guidelines for unresectable or recurred HCC with large diameters or multiple intrahepatic lesions even for portal vein tumor thrombus in some Asia-Pacific regions (19–23). Kim HC et al. (24) reported that selective chemoembolization *via* the caudate artery for solitary caudate lobe HCC is possible in most patients and a critical factor in longer overall survival and progression free survival. Won Seok Choi et al. (25) demonstrated that most tumor-feeding arteries supplying HCCs in the caudate lobe could be found by C-arm CT. Although many studies (18, 26–28) have reported the efficacy of TACE, RFA or combined treatment on HCC-CL, the reported tumor diameter or size is often relatively small, which is inconsistent with real-world data, because most HCC are already in the middle and advanced stages when they are diagnosed (29). Therefore, the present retrospective study was conducted to evaluate the effectiveness and safety of transcatheter arterial chemoembolization (TACE) for HCC arising or involving the caudate lobe found at initial presentation in the real world.

## Methods

### Study Design and Patient Selection

This was a retrospective study and performed according to the guidelines of the Helsinki Declaration. This study was approved by Ethics Committee of Tongji Medical College, Huazhong University of Science and Technology. The written informed consent was waived due to the retrospective nature of this study.

**Figure 1:**
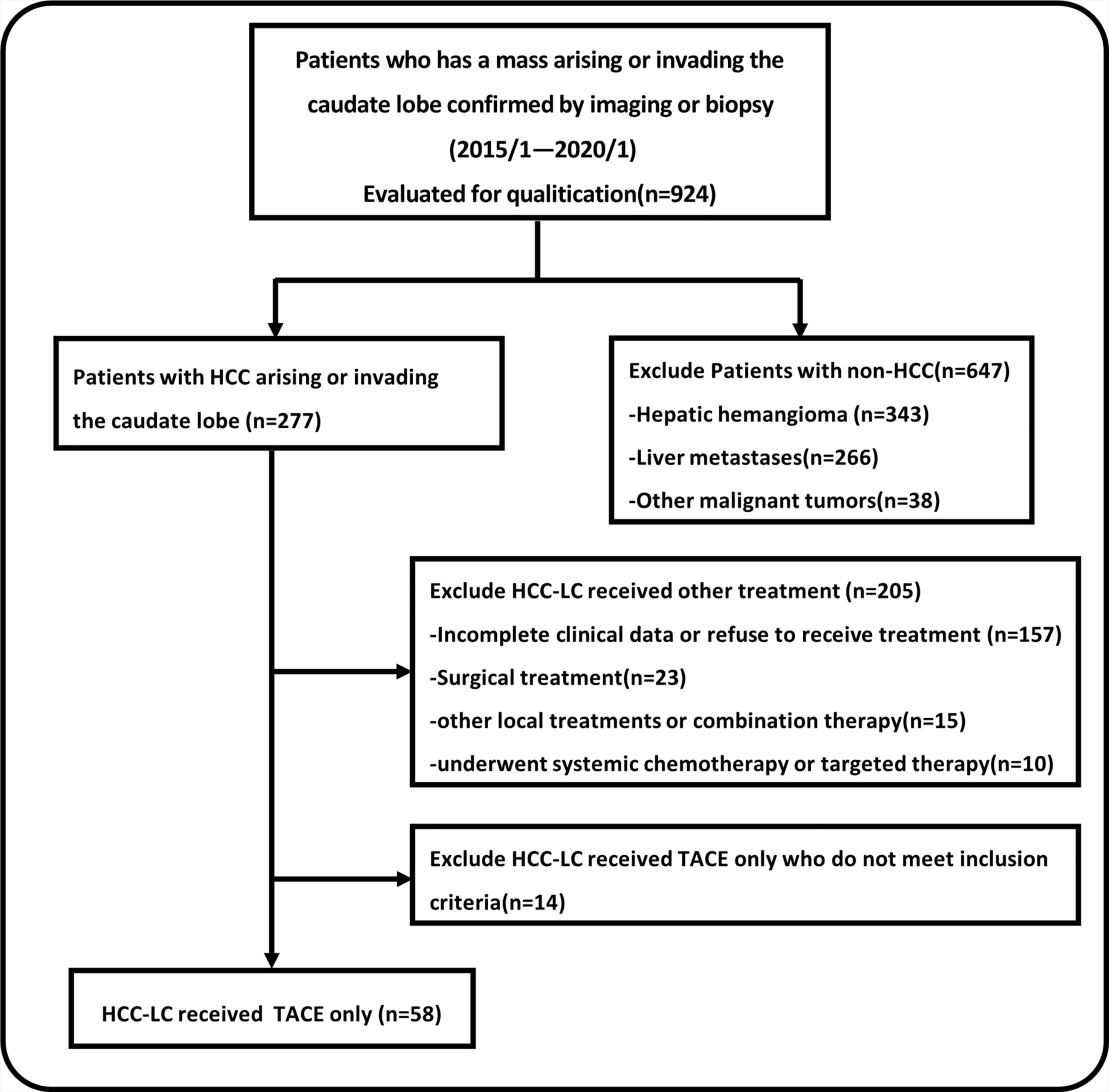
Patient enrolment and categorization flow chart.

As a result of searching radiologic database of our hospital, a total of 924 consecutive patients had a tumor arising or invading the caudate lobe confirmed by imaging or biopsy in our medical center between January 2015 and January 2020. We finally included 58 patients whose target lesions were in the caudal lobe or the caudal lobe of the liver was violated that could be measured at initial chemoembolization. The patient enrolment and categorization flow chart were shown in **Figure 1**.

The inclusion criteria were as follows: (1) HCC was measurable and unresectable or “ablation unsuitable” arising or involving the caudal lobe, which means the tumor was located near the inferior vena cava and peripheral gastrointestinal tract and had limited ablation or resection range; (2) Liver function status at Child-Pugh class A or B; (3) the Eastern Cooperative Oncology Group

(ECOG) performance status of 0 or 1; (4) No severe coagulopathy or ascites (e.g., platelets ≥50,000/ml, prothrombin time ratio ≥ 50%); (5) No previous treatment; (6) Available medical records. Exclusion criteria were: (1) Incomplete clinical data; (2) Tumor thrombus in the main portal vein, inferior vena cava; (3) Extrahepatic metastasis at preprocedural imaging study; (4) Severe liver dysfunction (Child-Pugh class C); (5) Patients were received other combination therapy (**Figure 1**).

### TACE Procedure

TACE was performed according to our institutional standard protocol and has been previously reported (30, 31). All operators had at least eight years of experience in performing TACE procedures. TACE was performed using transfemoral arterial access route with a micro-puncture system by placing a 5F vascular introducer (Cook, Bloomington, Indiana, USA) and celiac or superior mesenteric arteriography was carried out to assess the arterial anatomy, tumor supplying vessel and patency of the portal vein. A 2.6-Fr microcatheter (Terumo, Japan) was inserted into the tumor donor arteries as superselectively as possible to identify the staining and arteries feeding the target lesions. First, an emulsion of 5-20 mL lipiodol (Lipiodol Ultrafluido, Guerbet, France) mixed with 20-60 mg doxorubicin hydrochloride (Hisun Pharmaceutical Co. Ltd., Zhejiang, China) was injected into tumor feeding branch of the hepatic artery. Then the gelatin sponge particles (300–500 um, Cook, Bloomington, Indiana, USA) mixed with contrast material were administered into the tumor-feeding arteries until stasis of the arterial flow was achieved.

### Definition and Evaluation of Data

Selective chemoembolization was defined as using microcatheter systems to catheterize each tumor’s feeding blood vessel branches in segment or subsegment and transport chemotherapy drugs mixed with lipiodol-based regimens followed by embolic agents, and no residual tumor staining was present (32). Modified Response Evaluation Criteria in Solid Tumors (mRECIST) was used to evaluate treatment response by two interventional radiologists (33), that was carried out at the 1-1.5 month after the first TACE procedure and then every 2 or 3 months until the time of progression or death. Objective tumor regression (ORR) referred to (complete response) CR or PR (partial response). Disease control rate (DCR) was used to represent the portion of patients who reach CR+PR+SD (stable disease). The diagnosis of macroscopic vascular invasion (MVI) was based on standard radiological imaging prior to treatment based on liver vessel structure and prognosis for different location of vascular tumor thrombus, including portal and hepatic vein tumor and its branch thrombus (34). Overall survival (OS) and progression-free survival (PFS) were calculated for each patient from the date of the first TACE to the date of death or the last follow up, and to the date of tumor progression (intrahepatic recurrence or new intrahepatic or extrahepatic lesions developed) or the last follow up, respectively. When a residual or recurrent tumor was detected, decisions about additional treatment were made according to the recurrence pattern, underlying liver function, and overall clinical condition of the patient.

Adverse events and complications after therapy were identified and described according to the Society of Interventional Radiology Classification system for Complications by Outcome (35). Major complications were defined as events leading to death and disability.

### Follow-Up and Repeated TACE

All patients were followed up until October 2020. Follow-up contents included laboratory tests, contrast-enhanced computed tomography (CT) or magnetic resonance (MR) imaging examination were performed 4-6 weeks after the treatment. Once local progression or intrahepatic metastasis occurs, palliative TACE treatment was given “on demand” until it is intolerable. Imaging (contrast-enhanced CT or MR) and laboratory examinations were performed every 2–3 months for patients, follow-up continued until the patient died or the end point of this study’s follow-up.

### Statistical Analyses

All statistical analyses were performed by using SPSS software (version 24.0; IBM, Armonk, New York). Quantitative data were expressed as mean ± standard deviation (SD), while qualitative data were represented by proportion. OS and PFS were plotted by using Kaplan–Meier method. Calculate 95% confidence intervals (CI) for median OS, median PFS, and hazard ratio (HR). Univariate analysis was performed using log-rank test, in which variables with P less than 0.1 were included in the multivariate analysis, which was implemented with the Cox proportional hazard regression model. All statistical tests were two tailed, and P< 0.05 regarded as significant difference.

## Results

### Study Population and Patient Characteristics

From January 2015 to January 2020, a total of 924 patients have a tumor arising or invading the caudate lobe as a result of searching radiologic database of our hospital and 866 patients were excluded because they did not meet the study requirements, as shown in **Figure 1**. Among them, 277 patients were diagnosed as HCC that depended on the diagnostic criteria of the European Association for the Study of Liver (EASL) and the American Association for the Study of Liver Disease (AASLD) (11, 12). Prior to these patients undergoing initial TACE, the treatment strategy was recommended by the multidisciplinary tumor board. 157 cases were excluded because most cases were taken from the hospital outpatient system and imaging reports suggested the presence of HCC in the caudal lobe, but there was no hospitalization or the follow-up data missed. Finally, a total of 58 patients were included in this study, the detailed baseline clinical characteristics of the 58 patients were summarized in **Table 1**. In general, the majority of patients who underwent TACE had hepatitis B virus infection (81%), Child-Pugh A liver function (84.5%), with large tumor sizes (7.5 ± 4.0, range 2-16.5cm) and the middle and advanced patients (86.2% in total). Among these patients, 32 patients whose target lesions originated in the caudate lobe, and the tumors in the other 26 patients invaded the caudate lobe and were unresectable. Nearly half of patients with caudate lobe HCC have multiple blood supply (46.6%) and multiple tumors (44.8%). Superselective chemoembolization *via* a 2.6-Fr microcatheter was achieved in 49 (84.5%) of the 58 patients. Nonselective chemoembolization was performed in 9 (15.5%) patients because of failed catheterization of the tumor-feeding vessels that were not clearly indicated on digital subtraction angiography (DSA) and extraordinarily technical difficulty owing to the tumor-feeding vessels’ small caliber or the acute angulation. **Supplementary Table 1** showed that there was no significant statistical difference (P > 0.05) in baseline data between the two groups except gender and total bilirubin. It means that the two groups are comparable without selection bias. Repeated TACE could continue to be used “on demand” when the multidisciplinary tumor board considered that TACE was promising for the control of intrahepatic lesions, such as localized progression or metastasis in the liver. The average number of TACE sessions was 5 (1-15). The median follow-up period was 17.5 months (range, 1–65 months). Before the final follow-up, 49 (84.5%) patients had experienced varying degrees of disease progression. 21 (36.2%) patients were deemed TACE intolerable (extensive extrahepatic spread, diffuse liver metastasis), 15 received targeted drug therapy and 6 received the best supportive treatment in this circumstance. A total of 39 (67.2%) people died due to extensive metastases or liver failure during the observation period.

**Table 1:** Baseline clinical characteristics of patients.

Characteristic	Patients with TACE treatment (No, %; Mean ± SD)
**Gender**	
Male	42 (72.4%)
Female	16 (27.6%)
**Age (y)**	55.4 ± 12.1
**Hepatitis**	
Hepatitis B	47 (81%)
Other	11 (19%)
**Albumin (g/L)**	37.8 ± 5.2
**Total bilirubin (µmol/L)**	20.7 ± 15.8
**Platelet count (10^9^/L)**	175.7 ± 88.7
**ALT(IU/L)**	43.8 ± 31.6
**AST (IU/L)**	52.9 ± 41.7
**Prothrombin time(s)**	14.0 ± 1.9
**ECOG**	
0	54 (93.1%)
1	4 (6.9%)
**AFP (ng/ml)**	
>400	21 (36.2%)
≤400	37 (63.8%)
**Liver cirrhosis**	
Absent	26 (44.8%)
Present	32 (55.2%)
**Ascites**	
Absent	51 (87.9%)
Present	7 (12.1%)
**BCLC stage**	
A	8 (13.8%)
B	35 (60.3%)
C	15 (25.9%)
**Child-Pugh score**	
A	49 (84.5%)
B	9 (15.5%)
**Macroscopic vascular invasion**	
Yes	12 (20.7%)
No	46 (79.3%
**Tumor size (cm)**	
Mean ± SD	7.5 ± 4.0
Range	2-16.5
**Number of tumors**	
1	32 (55.2%)
>1	26 (44.8%)
**Tumor-feeding artery**	
Single	31 (53.4%)
Multiple	27 (46.6%)
**Superselective embolization**	
Yes	49 (84.5%)
No	9 (15.5%)
**TACE sessions**	
1	10 (17.2%)
2 or more	48 (82.8%)
**Tumor location**	
Spigel lobe	29 (50.0%)
Paracaval portion	23 (39.7%)
Caudate process	6 (10.3%)
**Origin of tumor/tumor distribution**	
CL	32 (55.2%)
R-CL	20 (34.5%)
L-CL	6 (10.3%)

SD, Standard deviation; ALT, Alanine aminotransferase; AST, Aspartate aminotransferase; PT, Prothrombin time; AFP, Alpha fetoprotein; ECOG, Eastern Cooperative Oncology Group; CL, Caudate lobe; R-CL, Right-Caudate lobe; L-CL, Left- Caudate lobe.

### Treatment Response and Complications

The treatment response at the first follow-up CT or MR was CR in 6 patients (10.3%), PR in 25 patients (43.1%), and SD in 15 patients (25.9%). ORR was 53.4% and DCR was 79.3%. No treatment-related deaths occurred in this study. Two patients had bile duct related complications, with an incidence of 3.4%. Percutaneous biliary intervention has been needed in symptomatic patients. Common minor complications occurred in 18 patients (31.0%), including 15 patients (25.9%) with fever, 13 patients (22.4%) with elevated total bilirubin, 7 patients (12.1%) with elevated serum alanine aminotransferase or aspartic aminotransferase levels, 4 patients (6.8%) with abdominal pain, 6 patients (10.3%) with nausea and vomiting. These symptoms lasted 2–7 days and were relieved by symptomatic treatment before discharge. No other serious complications occurred.

### Overall Survival and Progression-Free Survival Analysis

**Figure 2:**
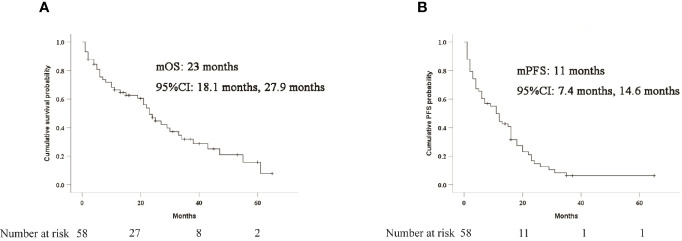
Kaplan-Meier curves of survival outcomes after TACE treatment in all patients.

**Figure 3:**
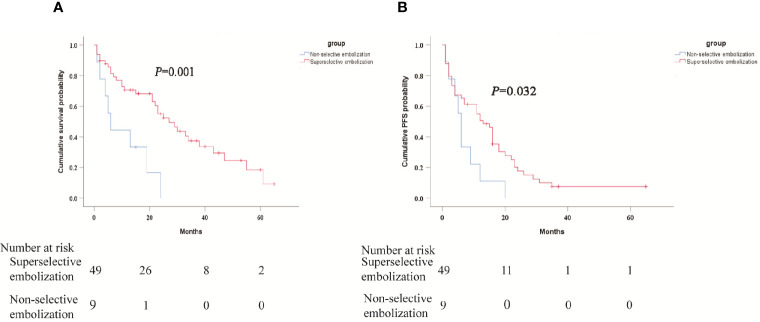
Kaplan-Meier curves of subgroup analysis survival outcomes by selective chemoembolization.

The median OS was 23 months (95%CI: 18.1-27.9). The median PFS was 11 months (95%CI: 7.4-14.6). The 1-, 3-, and 5-years OS rates were 66.5%, 31.9%, and 15.7%, respectively. The 0.5, 1-, and 3-years PFS rates were 60.3%, 44.5% and 6.3% respectively. Survival curves of patients were shown in **Figure 2**. The median OS was 27 months (95% CI 17.7 months, 36.3 months) for superselective chemoembolization in HCC-CL and 6 months (95% CI 3.1 months, 8.9 months) for non-selective chemoembolization (P = 0.001) (**Figure 3A**). Meanwhile, the median PFS in the two groups was 13 months (95% CI 8.1 months, 17.9 months) and 6 months (95% CI 4.6 months, 7.4 months), respectively (P = 0.032) (**Figure 3B**).

### Prognostic Factors Affecting OS and PFS

The risk factors for OS and PFS were analyzed in the **Table 2**. Multivariate analyses identified TBIL (HR = 1.033, 95% CI: 1.004–1.063, P = 0.027), BCLC stage (HR = 6.796, 95% CI: 1.200-38.482, P = 0.030), nonselective chemoembolization (HR = 5.512, 95% CI: 1.905-15.947, P = 0.002) and TACE session (HR = 3.998, 95% CI: 1.465–10.909, P = 0.007) as four significant factors associated with OS **(** **Table 3** **)**. Single tumor (HR = 0.439, 95% CI: 0.214–0.900, P = 0.025) was a unique factor for PFS in multivariate analysis **(** **Table 4** **)**.

**Table 2:** Univariate analysis of prognostic factors for overall survival (OS) and progression-free survival (PFS).

Variables	OS		PFS	
	HR (95% CI)	*P* value	HR (95% CI)	*P* value
**Gender**				
Male	1		1	
Female	0.954 (0.462, 1.970)	0.899	1.010 (0.544, 1.877)	0.974
**Age (y)**	0.975 (0.947, 1.003)	0.081	0.996 (0.971, 1.021)	0.729
**Hepatitis**				
Hepatitis B	1		1	
Other	0.575 (0.223, 1.482)	0.252	0.539 (0.241, 1.206)	0.133
**Albumin (g/L)**	0.970 (0.912, 1.030)	0.319	0.988 (0.936, 1.043)	0.662
**Total bilirubin (µmol/L)**	1.022 (1.001, 1.044)	0.045	1.012 (0.992, 1.033)	0.254
**Platelet count (10^9^/L)**	1.001 (0.996, 1.005)	0.759	1.001 (0.997, 1.004)	0.602
**ALT(IU/L)**	1.010 (1.001, 1.019)	0.038	1.008 (0.999, 1.017)	0.074
**AST (IU/L)**	1.006 (1.000, 1.012)	0.056	1.004 (0.999, 1.009)	0.105
**Prothrombin time, INR**	1.028 (0.890, 1.188)	0.703	0.936 (0.816, 1.072)	0.339
**ECOG**				
1	1		1	
0	0.464 (0.107, 2.023)	0.307	0.416 (0.123, 1.402)	0.157
**AFP (ng/ml)**				
>400	1		1	
≤400	0.462 (0.241, 0.885)	0.020	0.424 (0.231, 0.777)	0.006
**Liver cirrhosis**				
Present	1		1	
Absent	1.188 (0.626, 2.255)	0.598	1.255 (0.719, 2.191)	0.424
**Ascites**				
Present	1		1	
Absent	1.106 (0.429, 2.848)	0.835	1.699 (0.701, 4.118)	0.240
**BCLC stage**				
A	1		1	
B	2.365 (0.710, 7.872)	0.161	2.868 (1.110, 7.405)	0.030
C	5.970 (1.614, 22.089)	0.007	6.196 (2.056, 18.670)	0.001
**Child-Pugh score**				
B	1		1	
A	0.725 (0.317, 1.660)	0.447	1.145 (0.536, 2.446)	0.727
**Macroscopic vascular invasion**				
Yes	1		1	
No	0.334 (0.157, 0.710)	0.004	0.396 (0.193, 0.811)	0.011
**Tumor size (cm)**	1.089 (1.005, 1.179)	0.036	1.093 (1.017, 1.174)	0.016
**Number of tumors**				
** >1**	1		1	
** 1**	0.731 (0.387, 1.379)	0.333	0.504 (0.284, 0.893)	0.019
**Tumor-feeding artery**				
Multiple	1		1	
Single	0.976 (0.513, 1.854)	0.940	0.788 (0.453, 1.372)	0.400
**Superselective embolization**				
Yes	1		1	
No	3.530 (1.527, 8.163)	0.003	0.610 (0.347, 1.070)	0.085
**Child-Pugh score**				
A	1		1	
B	4.634 (1.346, 15.935)	0.015	2.156 (1.019, 4.562)	0.044
**TACE sessions**				
2 or more	1		1	
1	2.083 (0.939, 4.622)	0.071	1.232 (0.595, 2.550)	0.575
**Tumor location**				
Spigel lobe	1		1	
Paracaval portion	1.540 (0.771, 3.079)	0.221	1.547 (0.850, 2.814)	0.153
Caudate process	1.071 (0.362, 3.173)	0.901	1.261 (0.511, 3.109)	0.614
**Origin of tumor**				
CL	1		1	
R-CL	1.548 (0.789, 3.034)	0.203	1.513 (0.821, 2.789)	0.184
L-CL	1.350 (0.395, 4.609)	0.632	1.263 (0.483, 3.303)	0.634

HR, Hazard ratio; CI, Confidence interval; ALT, Alanine aminotransferase; AST, Aspartate aminotransferase; PT, Prothrombin time; AFP, Alpha fetoprotein; ECOG, Eastern Cooperative Oncology Group; CL, Caudate lobe; R-CL, Right-Caudate lobe; L-CL, Left- Caudate lobe.

**Table 3:** Multivariate analysis of prognostic factors for overall survival (OS).

Variables	HR (95% CI)	*P* value
**Age (y)**	0.980 (0.950, 1.010)	0.190
**Total bilirubin (µmol/L)**	1.033 (1.004, 1.063)	0.027
**ALT(IU/L)**	0.997 (0.985, 1.010)	0.653
**AST (IU/L)**	1.002 (0.993, 1.012)	0.640
**AFP (ng/ml)**		
>400	1	
≤400	0.610 (0.254, 1.467)	0.270
**BCLC stage**		
A	1	
B	6.796 (1.200, 38.482)	0.030
C	6.543 (0.377, 113.401)	0.197
**Macroscopic vascular invasion**		
Yes	1	
No	0.391 (0.046, 3.323)	0.390
**Tumor size (cm)**	1.007 (0.905, 1.121)	0.894
**Superselective embolization**		
Yes	1	
No	5.512 (1.905, 15.947)	0.002
**Child-Pugh score**		
A	1	
B	0.511 (0.139, 1.879)	0.312
**TACE sessions**		
2 or more	1	
1	3.998 (1.465, 10.909)	0.007

HR, Hazard ratio; CI, Confidence interval.

**Table 4:** Multivariate analysis of prognostic factors for progression-free survival. (PFS).

Variables	HR (95% CI)	*P* value
**ALT(IU/L)**	1.000 (0.989, 1.011)	0.974
**AFP (ng/ml)**		
>400	1	
≤400	0.521 (0.249, 1.089)	0.083
**BCLC stage**		
A	1	
B	2.275 (0.606, 8.542)	0.223
C	1.928 (0.246, 15.102)	0.532
**Macroscopic vascular invasion**		
Yes	1	
No	0.293 (0.052, 1.650)	0.164
**Tumor size (cm)**	1.020 (0.930, 1.120)	0.671
**Number of tumors**		
** >1**	1	
** 1**	0.439 (0.214, 0.900)	0.025
**Superselective embolization**		
Yes	1	
No	1.902 (0.817, 4.425)	0.136
**Child-Pugh score**		
A	1	
B	0.438 (0.163, 1.173)	0.100

HR, Hazard ratio; CI, Confidence interval.

### Subgroup Analysis

**Figure 4:**
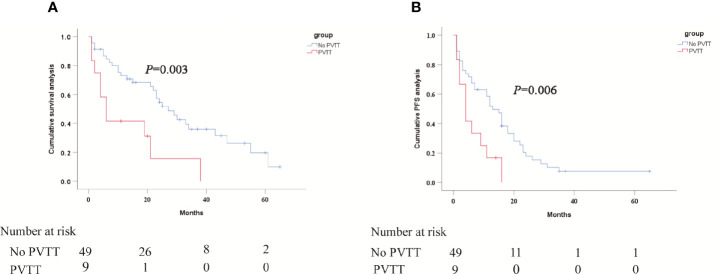
Kaplan-Meier curves of subgroup analysis survival outcomes by MVI.

**Figure 5:**
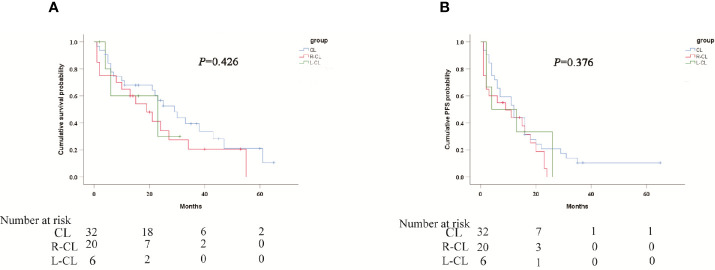
Kaplan-Meier curves of subgroup analysis survival outcomes by tumor distribution.

The median OS was 27 months (95% CI 19.5 months, 34.5 months) in patients without MVI and 6 months (95% CI 2.7 months, 9.3 months) in patients with MVI (P = 0.003), as shown in **Figure 4A**. Meanwhile, the median PFS was 13 months (95% CI 9.4 months, 16.6 months) and 4 months (95% CI 1.8 months, 6.2 months), respectively (P = 0.006) **(** **Figure 4B** **)**. The median OS was 29 months (95% CI 18.8 months, 39.2 months) for CL alone and 19 months (95% CI 9.4 months, 28.6 months) for R-CL,23 months (95% CI 0 months, 49.5 months) for L-CL, respectively (P = 0.426) **(** **Figure 5A** **)**. The median PFS was 12 months (95% CI 6.5 months, 17.5 months) for CL alone and 9 months (95% CI 0 months, 19.1 months) for R-CL, 4 months (95% CI 0 months, 17.2 months) for L-CL, respectively (P = 0.376) **(** **Figure 5B** **)**

## Discussion

Although there were a large number of studies confirming that surgery and percutaneous ablation treatment could bring survival benefits to some patients with HCC in the caudate lobe (2, 16, 17, 26), these treatment options remained difficult to achieve and had higher local recurrence rate owing to its deep location, adjacent major vessels and limited therapeutic margin (5, 7, 15, 36, 37). Our study demonstrated that in the real world, for some selected patients with good liver function and high tumor burden, TACE could have significant benefits of survival and disease control.

To our knowledge, there are currently a few studies confirming the efficacy of TACE in some patients with early-stage HCC-CL under the advances in microcatheters and DSA-guided devices (3, 24, 27, 28). Lu et al. (38) reported that early diagnosis and active treatment could result in long-term survival and HCC-CL had results similar to those of chemoembolization for HCC in other segments, while Terayama et al. (39) reported that chemoembolization for caudate lobe HCC was associated with a high local recurrence rate. Kim et al. (24) reported that the overall survival rates of patients with solitary HCC-CL with a median diameter of 2.5 cm undergoing initial TACE, at 3 and 5 years were 65% and 56%, which were comparable to those after surgical resection. Choi et al. (25) reported the use of C-arm CT enabled more accurate selective chemoembolization, which may result in lower cumulative local recurrence rates, but the tumor states of patients were usually small, not in line with clinical practice, so it cannot represent the vast majority of patients with caudate lobe liver cancer.

This study not only included patients whose tumor originating in the caudate lobe, but also patients whose caudate lobe was violated by the tumor of left and right lobes. In our study, there were more initially treated patients whose tumor burden was more than 5cm in diameter and had large blood vessels invaded, which was in consistent with the actual treatment process for HCC patients in the Asia-Pacific region. Similar to the baseline data of the population included in this study, the latest TACTICS trial (40) reported patients with unresectable HCC undergone TACE alone had a median PFS 13.5 months, comparable to this research 11.0 months. OS at 1 year was longer than that in this study (82.7% *vs*. 66.5%). A meta-analysis, including a total of 10,108 patients treated with conventional TACE, reported that the overall survival rates at 1,3, and 5 years were 70.3%, 40.4%, and 32.4%, respectively (20). Its long-term prognosis is better than this study, showing caudate liver cancer may have a worse prognosis, or baseline condition included in the study are worse.

More retrospective studies or prospective randomized controlled trials (RCT) comparing caudate and non-caudal liver cancers need to be carried out to verify this hypothesis.

Multivariate analysis showed that nonselective chemo-embolization, high TBIL, BCLC stage B and once TACE session seemed to have a greater impact on OS. Single tumor was a unique factor for PFS in multivariate analysis. It was confirmed by other researches (5, 7, 36) that attention should be not only paid to accurate super-selective intubation, but also to the number of tumors, liver function of patients, repeat TACE. Early combination of molecular targeted drugs may improve long-term efficacy.

The results of subgroup analyses showed that the application of superselective chemoembolization and the absence of portal vein invasion could have greater OS and PFS benefits regardless of whether it originated from the caudate lobe or invaded the caudate lobe. This was possibly due to mild chemoembolization would cause the increase of vascular endothelial growth factor (VEGF) and hypoxia inducible factor (HIF), cause tumor recurrence or TACE resistance/failure (22). At the same time, portal vein invasion may indicate a poor prognosis and the possibility of distant metastasis. More cases needed to be accumulated to confirm this part of the issue.

To note, the characteristics showed that most of the patients with a higher tumor burden in HCC-CL were given superselective TACE treatment. For unresectable caudate lobe liver cancer, the long-term effect is acceptable, but the cumulative recurrence rate in 3 years is as high as 93.7%. Repeated TACE treatment may have good prognostic value for survival.

This study had some limitations. First, this was a retrospective study, there may be some inevitable selection biases. Findings from this study should be further expanded to multicenter to obtain higher-level medical evidence. Second, due to included patients with HCC-CL were often inoperable or ablation treatment, there is a certain degree of heterogeneity. Better stratification for these patients was needed to be done in a bigger cohort study. Besides, it was worth noting that the analyzed patients were coming from a single center in China which was known to have a high incidence of hepatitis B virus (HBV) associated HCCs. Given most of them are intermediate and advanced HCCs, the final research results may not be applicable to other regions or countries. Meanwhile, the present study lacks a control group to compare it with other treatment modality, and further studies are needed.

## Conclusion

In conclusion, our study indicated that, superselective TACE treatment might be associated with better survival benefits in unresectable or “ablation unsuitable” HCC in the caudate lobe without MVI and adequate liver function, compared with the non-selective TACE group, and should be considered as an important reliable therapy for surgeons and interventional radiologists.

## Data Availability Statement

The raw data supporting the conclusions of this article will be made available by the authors, without undue reservation.

## Ethics Statement

The studies involving human participants were reviewed and approved by Ethics Committee of Tongji Medical College, Huazhong University of Science and Technology. The patients/participants provided their written informed consent to participate in this study.

## Author Contributions

LY, HZ, XK, and KQ collected the patients’ data. LY drafted the manuscript. LY, YR, and CZ revised the manuscript. KQ, YR, and LC analyzed and interpreted the data. CZ made substantial contributions to the conception of the work. LY, YR, and CZ made substantial contributions to the design of the work, and have revised the manuscript substantively. All authors contributed to the article and approved the submitted version.

## Funding

This work was supported by grant from National Nature Science Foundation of China (81873919 and 81701800).

## Conflict of Interest

The authors declare that the research was conducted in the absence of any commercial or financial relationships that could be construed as a potential conflict of interest.

## Publisher’s Note

All claims expressed in this article are solely those of the authors and do not necessarily represent those of their affiliated organizations, or those of the publisher, the editors and the reviewers. Any product that may be evaluated in this article, or claim that may be made by its manufacturer, is not guaranteed or endorsed by the publisher.

## Abbreviations

HCC, Hepatocellular carcinoma; CL, Caudate lobe; R-CL, Right-Caudate lobe; L-CL, Left-Caudate lobe; MVI, macroscopic vascular invasion; RFA, Radiofrequency ablation; BCLC, Barcelona Clinic Liver Cancer; TACE, Transarterial chemoembolization; ECOG, the Eastern Cooperative Oncology Group; mRECIST, Modified Response Evaluation Criteria in Solid Tumors; CR, Complete response; PR, Partial response; SD, Stable disease; PD, Progressive disease; ORR, Objective response rate;OS, Overall survival; PFS, Progression-free survival; CTCAE, National Cancer Institute Common Terminology Criteria for Adverse Events; CT, Computed tomography; MR, Magnetic resonance; AFP, Alpha-fetoprotein; SD, standard deviation; CI, confidence intervals; HR, hazard ratio; EASL, European Association for the Study of the Liver; AASLD, American Association for the Study of Liver Disease; DSA, Digital subtraction angiography; HBV, Hepatitis B virus; VEGF, Vascular endothelial growth factor; HIF, Hypoxia inducible factor.
