# Supplementary material for: Superselective Transarterial Chemoembolization for Unresectable or “Ablation Unsuitable” Hepatocellular Carcinoma in the Caudate Lobe: A Real World, Single-Center Retrospective Study
Source: Front Oncol. 2021 Oct 28;11:678847. doi: 10.3389/fonc.2021.678847 (PMC8581471; doi:10.3389/fonc.2021.678847)
Supplement: Supplementary file 1 [file Table_1.docx]

Supplementary Material

**Supplementary Table 1:** Baseline Characteristics of the Patients in the Non-selective embolization-group and Superselective TACE embolization-group

| **Characteristics** | **Non-selective embolization-group**  (N=9)  (No, %; Mean ± SD) | **Superselective**  **embolization-group** (N=49)  (No, %; Mean ± SD) | ***P* value** |
| --- | --- | --- | --- |
| **Gender** |  |  | 0.004 |
| Male | 3 (33.4%) | 39 (79.6%) |  |
| Female | 6 (66.6%) | 10 (20.4%) |  |
| **Age (years)** | 57.1±18.5 | 55.1±10.9 | 0.754 |
| **ECOG**  **performance** |  |  | 0.587 |
| 0 | 8 (88.9%) | 46 (93.9%) |  |
| 1 | 1 (11.1%) | 3 (6.1%) |  |
| **Hepatitis** |  |  | 0.513 |
| Hepatitis B | 8 (88.9%) | 39 (79.6%) |  |
| Other | 1 (11.1%) | 10 (20.4%) |  |
| **Child-Pugh score** |  |  | 0.691 |
| A | 8 (88.9%) | 41 (83.7%) |  |
| B | 1 (11.1%) | 8 (16.3%) |  |
| **BCLC stage** |  |  | 0.062 |
| A | 0 | 8 (16.3%) |  |
| B | 4 (44.4%) | 31 (63.3%) |  |
| C | 5 (55.6%) | 10 (20.4%) |  |
| **TB (µmol/L)** | 12.9 ± 3.8 | 22.1±16.8 | 0.001 |
| **Albumin (g/L)** | 38.1 ± 5.1 | 37.7±5.2 | 0.828 |
| **PT(s)** | 14.2 ± 1.2 | 14.0±2.0 | 0.753 |
| **AST (µmol/L)** | 62.2±33.2 | 51.2±43.2 | 0.473 |
| **ALT (µmol/L)** | 47.6±53.1 | 43.1±26.7 | 0.704 |
| **PLT** | 150.4±58.3 | 180.3±93.0 | 0.357 |
| **Tumor size (cm)** | 9.0±4.5 | 7.3±3.9 | 0.232 |
| **Tumor location** |  |  | 0.349 |
| S | 3 (33.3%) | 26 (53.1%) |  |
| P | 4 (44.4%) | 19 (38.8%) |  |
| T | 2 (22.2%) | 4 (8.1%) |  |
| **Origin of tumor/**  **tumor distribution** |  |  | 0.494 |
| CL | 5 (55.6%) | 27 (55.1%) |  |
| R-CL | 4 (44.4%) | 16 (32.7%) |  |
| L-CL | 0 | 6 (12.2%) |  |
| **Tumor number** |  |  | 0.980 |
| = 1 | 5 (55.6%) | 27 (55.1%) |  |
| ＞1 | 4 (44.4%) | 22 (44.9%) |  |
| **Tumor-feeding artery** |  |  | 0.890 |
| Single | 5 (55.6%) | 26 (53.1%) |  |
| Multiple | 4 (44.4%) | 23 (46.9%) |  |
| **TACE sessions** |  |  | 0.596 |
| = 1 | 1 (11.1%) | 9 (18.4%) |  |
| ＞1 | 8 (88.9%) | 40 (81.6%) |  |
| **α-Fetoprotein**  **level** |  |  | 0.342 |
| ＞400 ng/mL | 2 (22.2%) | 19 (38.8%) |  |
| ≤400 ng/ml | 7 (77.8%) | 30 (61.2%) |  |
| **Macroscopic vascular invasion** |  |  | 0.056 |
| Absent | 5 (55.6%) | 41 (83.7%) |  |
| Present | 4 (44.4%) | 8 (16.3%) |  |
| **Liver cirrhosis** |  |  | 0.451 |
| Absent | 3 (33.3%) | 23 (46.9%) |  |
| Present | 6 (66.7%) | 26 (53.1%) |  |
| **Ascites** |  |  | 0.309 |
| Absent | 7 (77.7%) | 44 (89.8%) |  |
| Present | 2 (22.3%) | 5 (10.2%) |  |

Note. TACE: Transcatheter arterial chemoembolization; SD: Standard deviation; ECOG: Eastern Cooperative Oncology Group; BCLC: Barcelona Clinic Liver Cancer; TB: Total bilirubin ；PT: Prothrombin time; AST: Aspartate aminotransferase; ALT: Alanine aminotransferase; PLT: Platelet
